# Stochastic approach for the material properties of reinforcing textiles for the design of concrete members

**DOI:** 10.1038/s41598-021-01032-9

**Published:** 2021-11-09

**Authors:** Sergej Rempel, Marcus Ricker, Tânia Feiri

**Affiliations:** 1 Faculty of Architecture and Civil Engineering, Hochschule Augsburg University of Applied Sciences, An der Hochschule 1, 86161 Augsburg, Germany; 2 Institute of Structural Engineering, Hochschule Biberach University of Applied Sciences, Karlstraße 11, 88400 Biberach, Germany

**Keywords:** Structural materials, Statistics, Civil engineering

## Abstract

Textile-reinforced concrete has emerged in recent years as a new and valuable construction material. The design of textile-reinforced concrete requires knowledge on the mechanical properties of different textile types as well as their reinforcing behaviour under different loading conditions. Conventional load-bearing tests tend to be complex, time-consuming, costly and can even lack consistent specifications. To mitigate such drawbacks, a standardised tensile test for fibre strands was used to characterise the material properties needed for the design of a textile-reinforced concrete member. The standardised tensile test uses a fibre strand with 160 mm length, which is cut out of a textile grid. For the sake of this study, an epoxy resin-soaked AR-glass reinforcement was considered. The results show that the textile reinforcement has a linear-elastic behaviour, and the ultimate tensile strength can be statistically modelled by a Gumbel distribution. Furthermore, the results indicate that the modulus of elasticity is not influenced by the length or the number of fibre strands. Therefore, the mean value attained from the standardised test can be used for design purposes. These findings are essential to derive an appropriate partial safety factor for the calculation of the design values of the tensile strength and can be used to determine the failure probability of textile-reinforced concrete members.

## Introduction

Textile-reinforced concrete is an innovative composite material that uses mesh-like reinforcements made of, for example, alkali-resistant glass (AR-glass), carbon or basalt. In contrast to ordinary steel reinforcements, textile reinforcements do not corrode. Therefore, concrete covers can be minimised, enabling the design and construction of slender concrete components. In the case of concrete components reinforced with textiles made out of carbon fibres, known as “carbon concrete”^1–3^, recent developments show that some of the most favourable mechanical properties, namely the high tensile strength and durability, have contributed to the growing acceptance of these type of structural solutions across the construction sector^4–12^. An example is the world's first carbon concrete bridge in Ebingen (Germany)^13^.

Normally, building owners need an individual approval (e.g., the “ZiE” in Germany) or a general approval (e.g., the European Technical Assessments) for the production and construction of textile-reinforced concrete structures. To grant such proof of usability^14^, building authorities may request extensive load-bearing tests to evaluate the ultimate limit state (ULS) and the serviceability limit state (SLS). These tests tend to be complex, time-consuming, costly, and can even lack consistent specifications^6,15,16^. Hence, provisions that support the design of structural components without further experimental testing would be valuable to structural designers.

Kulas and Rempel^17^ proposed a promising modelling approach for the bending design, which hardly differs from the conventional calculation of steel-reinforced concrete. The difference lays in the mechanical behaviour of distinct textile reinforcements. As opposed to steel reinforcement, AR-glass or carbon reinforcement has a linear-elastic behaviour without a pronounced yield plateau and has three to seven times higher ultimate tensile strengths^6,15,16^. Based on such mechanical properties, a standardised tensile test for fibre strands was proposed by Hinzen^18^. This standard tensile test has the advantage that the influences from the weaving structure on the material parameters, namely damages and distortions during weaving, are considered on the analysis of the fibre strand. It is relevant to stress that the material properties of an individual fibre are not significant for the design of reinforcement^19^. Note that, in this context, multiple fibres form a filament and multiple filaments compose a strand^20^.

The results from the standardised tensile test can be used to determine the design values for a textile reinforcement, as epoxy resin-soaked AR-glass reinforcement. These values refer to the statistical parameters of two relevant materials properties: (1) ultimate tensile strength and (2) modulus of elasticity. The statistical characterisation of these textile reinforcement properties is important for the calculation of failure probabilities of textile-reinforced concrete members and for the calculation of partial safety factors. As numerous scientific studies emphasise (e.g.,^21–27^), the use of statistical concepts supports a probability-based safety analysis, and consequently, addresses the rationale of uncertainty that is inherent to the existing variability in loads and resistance of structural components. Thus, a probabilistic-based reasoning is essential to derive new design provisions and/ or to improve existing ones for the design of structural components.

This paper discusses the derivation of statistical parameters for the characterisation of an epoxy resin-soaked AR-glass textile through the above-mentioned standardised tensile test. The test can be used for all epoxy resin-soaked fibre strands with a linear-elastic behaviour. The article is structured as follows: firstly, the experimental setting is described including the procedures to analyse the influence of the length and number of fibre strands. Then, the test results are presented and discussed, following a reflection on their usability. Finally, the conclusions and limitations of this study are addressed. The results of the experimental campaign presented in this paper were partially described in a German publication^15^.

## Carrying out the experiment

### Standardised tensile test for fibre strands

**Figure 1:**
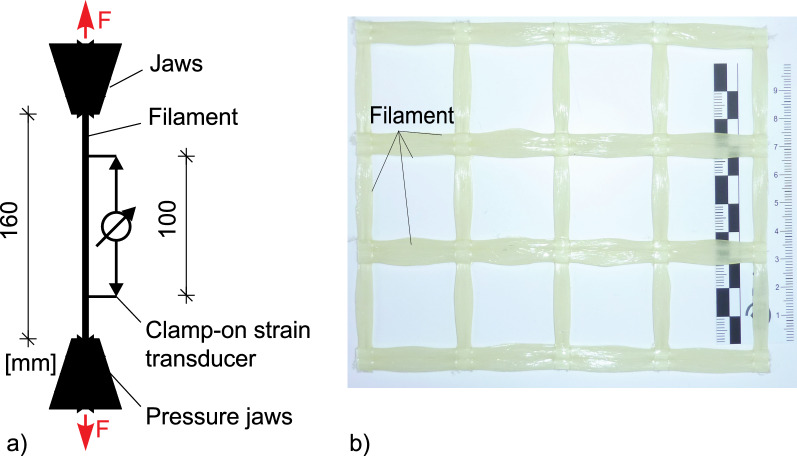
(**a**) Standardised tensile test set-up^17^. (**b**) Picture of the testing grid of AR-Glass reinforcement^15^.

The standardised tensile test proposed by Hinzen^18^ was used to determine the behaviour of textile reinforcement of an epoxy resin-soaked AR-glass textile. The test setup is schematically illustrated in Fig. 1a. The procedure starts with individual fibre strands being cut out of the soaked and cured textile layers placed in a textile grid (Fig. 1b). More precisely, a fibre strand with a length of 160 mm is cut out of the textile grid and a load is introduced through clamping jaws.

In addition to the load measurement, the strain is registered with two clamp-on strain transducers over a length of 100 mm. During the test, the fibre strand may break prematurely near the loading point. In such cases, the ultimate tensile strength of the fibre strands is not fully reached, and therefore, the individual value must not be considered. In most tensile tests, the extensometer shall be removed shortly before failure to prevent damage to the transducer. It is important to highlight that the strain can be measured with an optical measuring device. However, besides not being always available, the follow-up evaluation of the results can be rather complex^17^.

The above-described setup may be only used for fibre strands in which the load is transmitted through a stiff impregnation as it is the case of epoxy resin-soaked textiles. With these completely soaked fibre strands, the ultimate tensile strength, the modulus of elasticity obtained through the standardised tensile tests and the tests on composite components are similar (see Sect. “Influence of the number of fibre strands”). For this reason, the material parameters can be assessed on a pure fibre strand with a clamp-on strain transducer, and then, transferred to the composite component. With unimpregnated or only partially impregnated fibre strands, the load is transferred by the friction of the inner strands. The contact pressure of the clamping jaws increases the frictional stress so that more strands are directly involved in the load transfer. As a result, the tensile stresses obtained in the textile by means of a standardised tensile test are higher than those obtained, for example, through a bending test.

To determine a meaningful probability density function for the ultimate tensile strength of the textile reinforcement, multiple standardised tensile tests are required. To this, additional tests were conducted by industry partners. To avoid biased results, the test procedures were defined in advance alongside the collaborative setting between the partners. The test results were recorded in a shared database.

### Influence of the length of the fibre strand

The standardised tensile test was used to investigate the influence of the length of the fibre strand. During the test, the lengths considered increased gradually: 60 mm, 160 mm, 320 mm, and finally, 640 mm.

### Influence of the number of fibre strands

**Figure 2:**
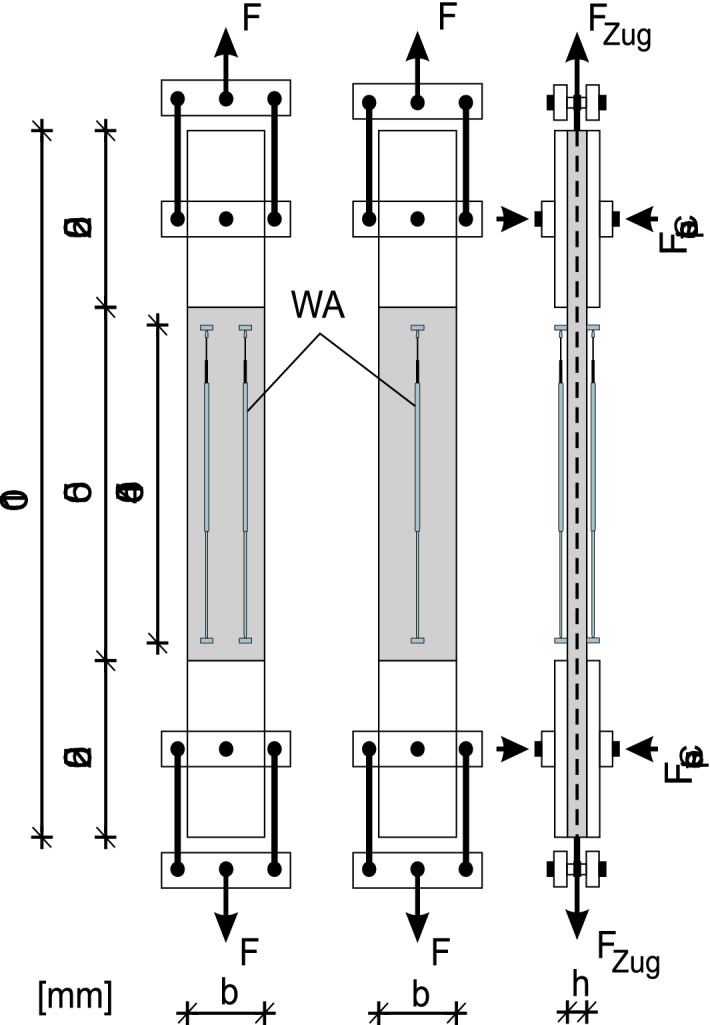
Schematic representation of the uniaxial tensile test on textile-reinforced concrete members^15^.

To investigate the influence of the number of fibre strands on the ultimate tensile strength of the reinforcement, fibre strands must be uniformly stressed. An additional requirement is that several fibre strands must be drawn at the same time. However, this is not possible with the proposed standardised tensile test. For this reason, the tensile test on a composite member was carried out, as it is shown in Fig. 2.

In contrast to the standardised tensile test, the tension load was not directly applied on a strand, but instead on a reinforced concrete body. The load was transferred from the testing machine to the composite member through the pressure jaws. This procedure ensured that the fibre strands were evenly loaded. In addition to the load, the strain was also recorded with linear variable differential transformers (LVDTs) over a reference length of 450 mm. This experimental setup is based on the RILEM recommendations^28^. A total of seven tensile test series were conducted on composite members reinforced with different numbers of fibre strands. To ensure that the ratio of the concrete cross-sectional area and the fibre strands remained roughly the same, the width *b* and the thickness* h* of the tensile specimen were adjusted. In addition, the standardised tensile tests on the fibre strands were conducted to show the transferability from a tensile test on the pure textile to an uniaxial tensile test of the composite material.

## Experimental results

### Standardised tensile tests on fibre strands

#### Material parameters for the design

**Figure 3:**
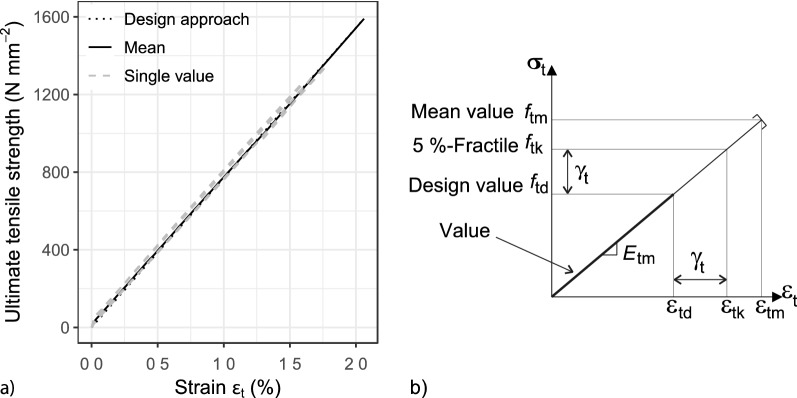
Stress–strain diagrams^15^ (**a**) AR-Glass reinforcement. (**b**) Design of textile-reinforced components.

The results of the standardised tensile tests are shown in the stress–strain diagram in Fig. 3a. An idealised stress–strain relationship is derived from the measurements, which can be later used for cross-sectional design of a textile-reinforced concrete component. The black curve represents the mean course of the individual experiments, which are indicated in grey. The dashed line in the figure refers to the idealised stress–strain relationship. The textile stress ${\upsigma }_{{\text{t}}}$ is calculated from the measured force *F* and the accumulated fibre strands cross sectional area *A*_r_ according to Eq. (1).

1 $$\sigma_{{\text{t}}} = { }\frac{{\text{F}}}{{A_{{\text{r}}} }}$$

The lines show an almost linear-elastic course until the failure point. This confirms the assumption that the fibre strands are practically stretched and hardly influenced by the knitting thread.

By using the results of the standardised tensile test, the material behaviour of the fibre strands with a linear-elastic approach can be determined with Eqs. (2) and (3). In principle, only two of the following parameters are required for the characterisation of the textile reinforcement behaviour since they have a relationship between them:

- $E_{{{\text{tm}}}} :{ }$ mean value of the modulus of elasticity (or Young’s modulus)
- $f_{{{\text{t}},{\text{u}}}} :{ }$ ultimate tensile strength
- $$\varepsilon_{{{\text{t}},{\text{u}}}} :{ }$$ ultimate strain

By assuming such linear-elastic behaviour, as it is illustrated in Fig. 3b, the textile stress value of each strain (Eq. 2) and the strain value of each stress (Eq. 3) can be determined for each point of the stress–strain diagram by using the mean value of the modulus of elasticity.

2 $$\sigma_{{\text{t}}} = \varepsilon_{{\text{t}}} \cdot E_{{{\text{tm}}}} \le \, f_{{{\text{t}},{\text{u}}}}$$

3 $$\varepsilon_{{\text{t}}} = \frac{{\sigma_{{\text{t}}} }}{{E_{{{\text{tm}}}} }} \le \, \varepsilon_{{{\text{t}},{\text{u}}}}$$

The measurement of the strain can be stopped at around 60% of the failure load to preserve the LVDTs from damage. To determine the 60% of the failure load, preliminary tests were conducted working as a reference for the remaining experimental campaign. This procedure was possible since the tests clearly show that the textile reinforcement behaves in a linear-elastic manner until it breaks, and therefore, the modulus of elasticity was practically constant.

#### Material parameters for the design approach

The material parameters are usually described through distribution functions, which are characterised by statistical parameters (or moments). The robustness of a distribution function strongly depends on the extent of representative data or, in this case, measurements. For this reason, several hundred standardised tensile tests were conducted. Since it has been assumed that ultimate tensile strengths are normally distributed without further appraisals^29^, the test results were used to evaluate the normality assumption.

**Figure 4:**
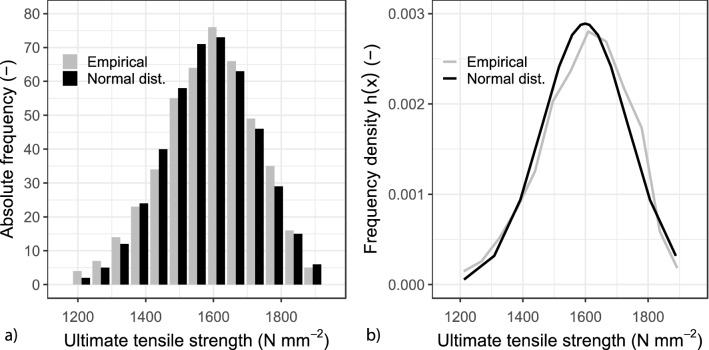
Statistical evaluation of the ultimate tensile strength^15^ (**a**) Histogram for the AR-Glass reinforcement (empirical and theoretical values). (**b**) Probability density function of the AR- Glass reinforcement.

The measured ultimate tensile strengths were divided into classes and then compiled in a histogram. The results of the AR-glass textiles are shown in Fig. 4a. A close observation of this figure leads to an immediate exclusion of some distribution families. The values were then converted into a frequency density $h\left( {\it{x}} \right)$ by generating the ratio of the relative frequency to the class width. If points are now placed in the mean values of the classes and these are connected, a curve of the frequency density is obtained, as it is illustrated in Fig. 4b. The shape of this curve provides a rough indication of the most suitable distribution family. In this case, the curve plotted in Fig. 4b resembles the probability density function of a Normal distribution. The expected value was approximated by the arithmetic mean value $\mu_{{\text{X}}} \approx \overline{x}_{{\text{X}}} = 1\;590\;{\text{N}}\;{\text{mm}}^{ - 2}$ and the standard deviation was estimated by the empirical standard deviation $\sigma_{{\text{X}}} \approx s_{{\text{X}}} = 138\;{\text{N}}\;{\text{mm}}^{ - 2}$. The mean value $\overline{x}_{{\text{X}}}$ and the empirical standard deviation $s_{{\text{X}}}$ were calculated from more than 400 experiments and then used in Eq. (4), which represents the probability density function of a Normal distribution^30^.

4 $$f\left( x \right) = \frac{1}{{\sigma_{X}\cdot \sqrt {2\pi } }} \,\exp \left( { - \frac{{\left( {x \,-\, \mu_{X} } \right)^{2} }}{{2\cdot \sigma_{X}^{2} }}} \right) = \frac{1}{{138\cdot \sqrt {2\pi } }}\, \exp \left( { - \frac{{\left( {x\, -\, 1\,590} \right)^{2} }}{{2\cdot \left( {138} \right)^{2} }}} \right)$$

It is important to highlight that the graphical plot alone does not confirm the assumption that the ultimate tensile strength is normally distributed. Therefore, a *Chi-Square* (${\upchi }^{2} )$ test was performed to test the data normality. This test starts with the definition of two hypotheses: *H*_0_ and *H*_1_. The so-called null hypothesis *H*_0_ states that the ultimate tensile strength is normally distributed, and *H*_1_ expresses the opposite^31^. In this test, the expected outcome frequencies and the observed outcome frequencies are compared (Fig. 4a). If the difference χ^2^ between the functional value is greater than the critical value ${\upchi }_{{\left( {1 - {\upalpha }} \right);{\upupsilon }}}^{2}$, the null hypothesis is rejected. This critical value was calculated for a significance level of α = 5%, which corresponds to the (1 − α) = 95% fractile of the ${\upchi }^{2}$ distribution with the associated degree of freedom ν = 12. In the χ^2^ test, the difference obtained is χ^2^ = 9, which is below the critical value ${\upchi }_{{\left( {1 - 0.05} \right);12}}^{2} = 21$.

The tests conducted do not reject that the ultimate tensile strengths of the fibre strand soaked with epoxy resin can be described by a Normal distribution function with satisfactory accuracy. This has been observed in various textile variants for both directions: warp and weft. Based on these conclusions, the arithmetic mean value, and the empirical standard deviation (i.e., the most common statistical estimators) were used to approximate the same statistical parameters of a Normal distribution. This describes the random character of the ultimate tensile strength. The entire set of test results cannot be presented in this paper due to space limitations; however, the results are available in^16^ for consultation.

### Influence of the length of the fibre strand

#### Experimental investigations

**Figure 5:**
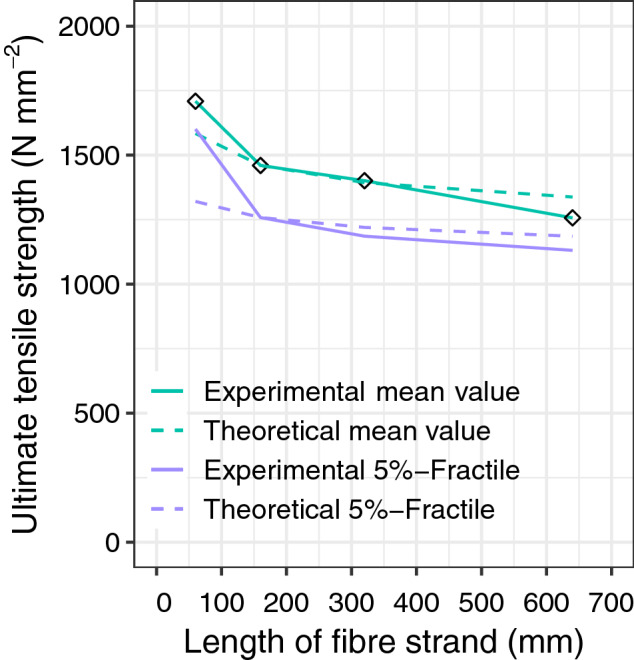
Ultimate tensile strength of the textiles depending on the length of the fibre strands.

The influence of the length of the fibre strand on the ultimate tensile strength was investigated for the four lengths of the AR-glass textile (see Sect. “Influence of the length of the fibre strand”) placed in the weft direction. Each length was tested at least seven times. The results were statistically evaluated under the assumption of a Normal distribution. These are summarised in Fig. 5, where the influence of the length on the ultimate tensile strength can be clearly observed. The mean value decreases non-linearly with an increasing length of the fibre strands. The fibre strand with a length of 60 mm—the shortest length—registered an average ultimate tensile strength of 1 709 N mm^-2^. On the other extreme, the tensile strength of a fibre strand with a length of 640 mm registered an average ultimate tensile strength of 1 257 N mm^−2^.

The authors believe that such difference can be justified by the scale effect, in which the number of imperfections increases with a growing length of the fibre strand. This scale effect was previously investigated by Griffith^32^. Among other relevant findings, Griffith observed that tensile strength decreased with increasing fibre length. More recently, extensive studies on the general issue of size effects have been conducted by Bažant ZP (e.g.,^33–35^). Additionally, Rypl^36^ and Chudoba^37^ found that the standard deviation decreases with an increasing length of fibre strands.

**Figure 6:**
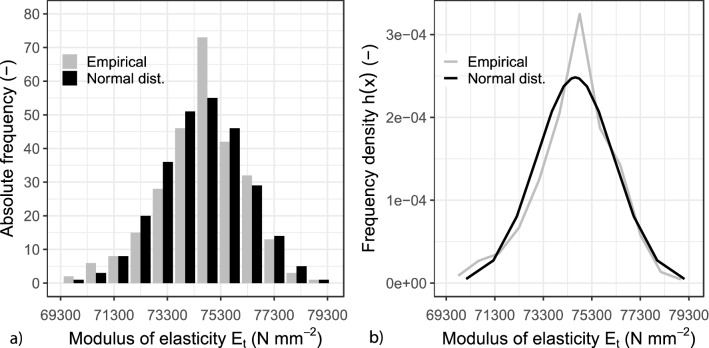
Statistical evaluation of the modulus of elasticity (**a**) Histogram for the AR-Glass reinforcement (observed values and expected values). (**b**) Probability density function of the AR- Glass reinforcement.

The influence of the fibre strand length on the modulus of elasticity was investigated for the four lengths of the AR-glass textile (see Sect. “Influence of the length of the fibre strand”) placed in the weft direction. Each length was tested at least seven times. The results were statistically evaluated under the assumption of a Normal distribution. These are summarised in Fig. 6. Figure 6a shows the histograms for AR glass textiles with the absolute frequencies. The grey bars represent the measured empirical values, the black bars represent the expected absolute frequencies. The frequency density curve obtained is illustrated in Fig. 6b, resembling a probability density function of a Normal distribution. In this curve, the expected value was approximated by the arithmetic mean value ${\upmu }_{{\text{X}}} \approx \overline{x}_{{\text{X}}} = 74\;618\;{\text{N}}\;{\text{mm}}^{ - 2}$ and the standard deviation was estimated by the empirical standard deviation ${\upsigma }_{{\text{X}}} \approx s_{{\text{X}}} = 1\;610\;{\text{N}}\;{\text{mm}}^{ - 2}$. The mean value $\overline{x}_{{\text{X}}}$ and the empirical standard deviation $s_{{\text{X}}}$ were calculated using Eq. 5, which represents the probability density function of a Normal distribution^30^, as previously mentioned.

5 $$f\left( x \right) = \frac{1}{{\sigma_{X} \cdot \sqrt {2\pi } }} \, \exp \left( { - \frac{{\left( {x\,-\,\mu_{X} } \right)^{2} }}{{2 \cdot \sigma_{X}^{2} }}} \right) = \frac{1}{{1\,610 \cdot \sqrt {2\pi } }} \, \exp \left( { - \frac{{\left( {x\, -\, 74\,618} \right)^{2} }}{{2 \cdot \left( {1\,610} \right)^{2} }}} \right)$$

The normality assumption was also evaluated with a *Chi-Square* test (χ^2^), where the expected outcome frequencies and the observed outcome frequencies are compared. The test indicated that for a significance level of ${\upalpha }$ = 5%, the existing values χ^2^ are always below the permissible critical value ${\upchi }_{{\left( {1 - 0.05} \right);12}}^{2}$. The entire set of test results cannot be not presented in this paper due to space limitations; however, the results are available in^16^ for consultation. The results confirm that the modulus of elasticity of fibre strands soaked with epoxy resin can be described by a Normal distribution function with satisfactory accuracy. Thus, similarly to the previous analysis, the arithmetic mean value and empirical standard deviation/variance were used to determine the statistical parameters of the Normal distribution (i.e., the expected value and the standard deviation/variance).

#### Theoretical investigations

The statistical background must be known for the conception of a design model and for the calculations needed in a reliability analysis. To derive conclusions about the fibre strand length, only the results of the standardised tensile tests (i.e., fibre strand length = 160 mm) are needed. To this, in this section, theoretical investigations are conducted to determine the statistical parameters required by means of mathematical calculations.

In this analysis, it was assumed that the fibre strands are successively connected in series and the ultimate tensile strength of each element was characterised by a normally distributed random variable *X*. By using the extreme value theory, it is possible to determine not only the expected value and the standard deviation of the ultimate tensile strength of a single fibre strand, but also the distribution function of multiple fibre strands connected in series. The fibre strands connected in series can be compared to a chain where if one element fails, a total failure of the system will occur. In this case, it was assumed that the weakest link governs the failure. Thus, the distribution of the minimum ultimate tensile strength that governs the series system, the so-called minimum $M_{{\text{n}}}$, can be determined through an extreme value theory. For any number of fibre strands *n*, the distribution function of the minimum $\it F_{{M_{n} }} \left( {\text{x}} \right)$ can be calculated through Eq. 6^31^. To this, it is used the cumulative distribution function $\it F_{{\text{X}}} \left( {\text{x}} \right)$ of the ultimate tensile strength, which can be derived from the results of the standardised tensile tests on a fibre strand with a length of 160 mm.

6 $${\text{P}}\left( {M_{{\text{n}}} \le {\it{x}}} \right) = F_{{M_{{\text{n}}} }} \left( {\it{x}} \right) = 1 - { }\left[ {1 - F_{{\it{x}}} \left( {\it{x}} \right)} \right]^{n}$$

Equation (6) is only valid for independent and identically distributed random variables with a cumulative distribution function $\it F_{{\text{X}}} \left( {\text{x}} \right)$^31^. This is the case of fibre strands connected successively one behind the other since each link is assumed to have the same distribution function. By derivating Eq. (6), the probability density function $\it f_{{M_{{\text{n}}} }} \left( {\text{x}} \right)$ of the minimum ultimate tensile strength $M_{{\text{n}}}$ can be determined through Eq. (7):

7 $$f_{{M_{{\text{n}}} }} \left( {\it{x}} \right) = f_{{\text{X}}} \left( {\it{x}} \right) \cdot n \cdot \left[ {1 - F_{{\text{X}}} \left( {\it{x}} \right)} \right]^{n - 1}$$

The fractile values of the extreme value distribution can be calculated by rearranging Eq. (6):

8 $$F_{{M_{{\text{n}}} }} \left( {x_{{\text{p}}} } \right) = 1 - { }\left[ {1 - F_{{\text{X}}} \left( {x_{{\text{p}}} } \right)} \right]^{n} = p$$

9 $$F_{X} \left( {x_{{\text{p}}} } \right) = 1 - \sqrt[n]{1 - p}$$

If the tensile strength of each link is assumed normally distributed, the fractile values of the extreme value distribution can be calculated according to Eq. (10):

10 $$x_{{\text{p}}} = F_{{\text{X}}}^{ - 1} \left( {x_{{\text{p}}} } \right) = \mu_{{\text{X}}} + \sigma_{{\text{X}}} {\Phi }^{ - 1} \left( {1 - \sqrt[n]{1 - p}} \right)$$

**Figure 7:**
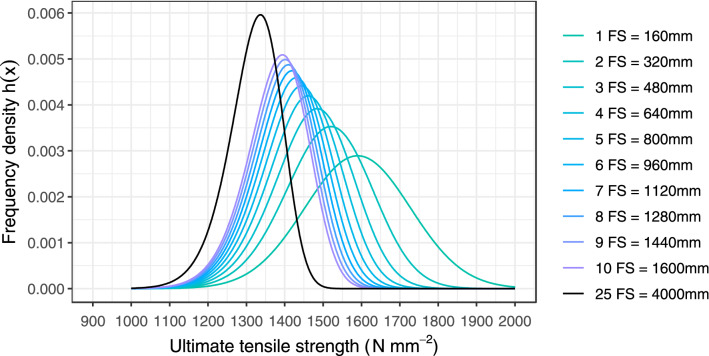
Probability density function of the ultimate tensile strength for a different number of fibre strands (FS) (adapted from^15^).

The densities of the extreme value distributions of different fibre strand lengths were calculated with Eq. (7) and compared in Fig. 7. This figure shows that as the fibre strand length increases, the expected value and the standard deviation of the extreme value distribution decrease. This trend is illustrated by the increasing slenderness of the curves and the shift to the left. The density of the extreme value distribution for the ultimate tensile strengths of *n* = 25 fibre strands connected in series is also visible in the black line of Fig. 7. This density is neither symmetrical nor normally distributed. For the convergence of the distribution, it was assumed that a generalised extreme value distribution type-I (Gumbel distribution)^38^ might be used. A Gumbel distribution has the advantage to facilitate the calculations in reliability assessments. This distribution is characterised by two parameters: *a* and *u*. The probability density function of this distribution for data minimum is described as follows:

11 $$f\left( x \right) = a \cdot e^{{a \cdot \left( {x\, -\, u} \right) - e^{{a \cdot \left( {x \, - \,u} \right)}} }}$$

To approximate the extreme value distribution by a Gumbel distribution according to Eq. (7), the ultimate tensile strengths of the 50%-Fractile (median) and the 5%-Fractile of the extreme value distribution are determined. Then, these values are assumed for the 50%-Fractile and the 5%-Fractile of the Gumbel distribution, respectively. The parameters *a* and* u* of the Gumbel distribution can then be determined according to Eqs. (12) and (13).

12 $$a = \frac{2.60368}{{F_{{M_{n} }}^{ - 1} \left( {0.05} \right) - F_{{M_{n} }}^{ - 1} \left( {0.50} \right)}}$$

13 $$u = 1.14077 \cdot F_{{M_{n} }}^{ - 1} \left( {0.50} \right) - 0.14077 \cdot F_{{M_{n} }}^{ - 1} \left( {0.05} \right)$$

with $F_{{M_{n} }}^{ - 1} \left( {0.50} \right)$ corresponding to the 50%-Fractile, $F_{{M_{n} }}^{ - 1} \left( {0.05} \right)$ being the 5%-Fractile of the extreme value distribution according to Eq. (6)*,* and *n* being the number of fibre strands.

**Figure 8:**
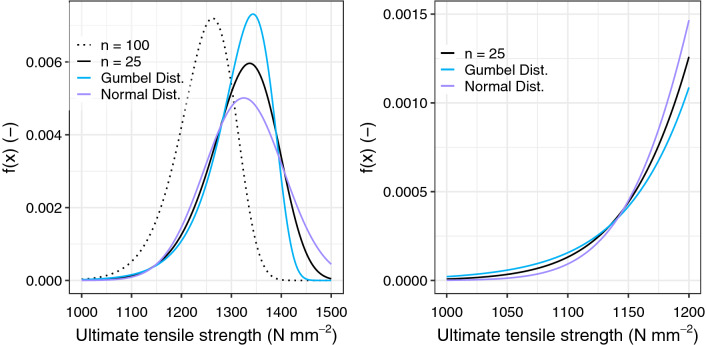
Probability-Density-Function of the extreme-value function and the approximation through a Normal and a Gumbel distribution functions (**a**) entire distribution and (**b**) selected area of the distribution at the tails.

Figure 8 shows the probability density functions of the extreme value function for two different numbers of fibre strands *n*: *n* = 25 and *n* = 100. For each *n*, the probability density function is approximated by a Normal distribution and by a Gumbel distribution. In Fig. 8a it can be observed that an approximation by a Gumbel distribution is more suitable with an increasing number of *n* (see curve for *n* = 100). An approximation by a Normal distribution lies slightly below the curve of the extreme value distribution.

Particularly relevant for the evaluation of failure probabilities is the behaviour of the Gumbel and the Normal distribution at the tails of the functions (Fig. 8b). Table 1 shows that for fractile values smaller than 2%, the Gumbel distribution lies slightly above the extreme value distribution, whereas the Normal distribution presents lower values. This trend can be seen in Fig. 8b, where the Normal distribution curve moves its course to below the extreme distribution curve at an ultimate tensile strength of roughly below 1 150 N mm^-2^. Such tail behaviour is not particularly surprising since, in theory, Normal distributions are characterised by thinner tails than those from extreme value distributions. Based on the values assessed, it can be stated that an approximation through a Normal distribution leads to underestimated values of failure probabilities, which in reliability analyses can be problematic. For very low failure probabilities, a Gumbel distribution is on the safe side.

**Table 1:** Extreme value distribution approximated by a Normal distribution and a Gumbel distribution for selected fractile values and for *n* = 25 fibre strands.

Values of *x*	Extreme value distribution	Normal distribution	Gumbel distribution
(Eq. 9)	(Eq. 7)	(Eq. 10)	(Eq. 11)
1 194	0.001117997	0.001295237	0.000968691
1 155	0.000491684	0.000513211	0.000458602
1 128	0.000260448	0.000232212	0.000268775
1 046	0.000030221	0.000010734	0.000053440
974	0.000003385	0.000000301	0.000012828

The values of *x* correspond to the 5%, 2%, 1%, 0.1%, and 0.01% values of the original extreme value distribution.

For design purposes, the 5%-Fractile is a governing value^39,40^, as it is also used as the characteristic tensile strength of the textile reinforcement $f_{{{\text{t}},{\text{k}}}}$. The design value of the tensile strength $f_{{{\text{t}},{\text{d}}}}$ is determined by dividing the characteristic value $f_{{{\text{t}},{\text{k}}}}$ by the partial safety factor $\gamma_{{\text{t}}}$ (Eq. 14). With a partial safety factor $\gamma_{{\text{t}}} = 1.0$, the characteristic value would be the same as the design value.

14 $$f_{{{\text{t}},{\text{d}}}} = { }\frac{{f_{{{\text{t}},{\text{k}}}} }}{{\gamma_{{\text{t}}} }}$$

### Influence of the number of fibre strands

#### Experimental investigations

**Figure 9:**
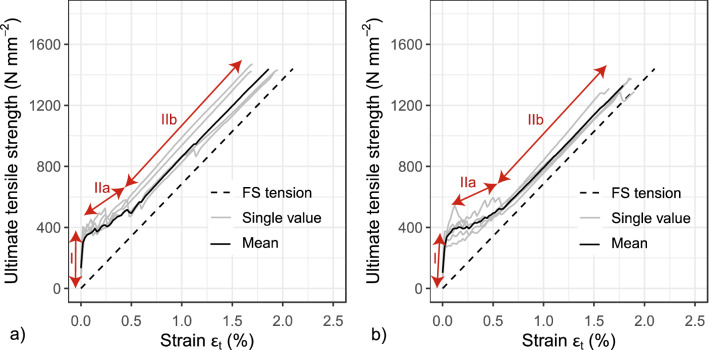
Tension-Strain diagram^15^: (**a**) two and (**b**) eight embedded fibre strands.

The influence of the number of fibre strands on the ultimate tensile strength was investigated with uniaxial tensile tests on composite members. In total, eight series with five tests (beginning with one fibre strand and ending with eight) were performed. The results are illustrated in the stress–strain diagrams of Fig. 9. In the tensile tests of the composite members, a textile failure always occurs. The textile tension $\sigma_{{\text{t}}}$ was calculated with Eq. (1) by considering the measured force *F* and the accumulated filament cross sectional area *A*_r_. Figure 9 displays an example of the stress–strain curve of an AR-glass textile. Figure 9a shows that the test sample was reinforced with only two fibre strands and Fig. 9b shows the results with eight fibre strands installed. The black curve represents the mean course of the individual experiments. The curves of the individual experiments are shown in grey. Figure 9 also shows that during the experiment three states occur: State I (uncracked), State IIa (crack formation) and State IIb (stabilised crack phase). In State IIb, the curve does not flatten, but runs parallel to the results of the standardised tensile test on the plain fibre strand, which is illustrated as dashed lines. In both tests, the same modulus of elasticity for the textile is achieved in State IIb. This supports the assumption that the test setup can be used to determine the influence of the number of fibre strands. Simultaneously, it can be concluded that the modulus of elasticity is not influenced by the number of fibre strands.

#### Theoretical investigations

The experimental investigations show that the number of fibre strands does not influence the mean value of the modulus of elasticity. This finding cannot be transferred to the ultimate tensile strength, since for each number of fibre strands, the tensile strength follows a distribution function with different parameters. Like in the analysis in Sect. “Theoretical investigations”, only the results of the standardised tensile test on an individual fibre strand are required to derive a possible mathematical relationship for any number of fibre strands. For this purpose, it is considered that the fibre strands (length = 160 mm) are virtually and successively connected next to one another. To each element, it is considered a Normal distribution, which was determined with the standardised tensile test for a single strand (see Sect. “Material parameters for the design approach”).

The fibre strands connected next to one another can be compared to a parallel connection where, in principle, if one element fails, a total failure occurs providing that no redistribution of stresses occurs. In contrast to steel that is characterised by a ductile failure behaviour, a brittle failure occurs as soon as the end of the linear-elastic range is reached. During the test, each fibre strand in the parallel system is loaded with the same load; however, the strand does not have the same ultimate tensile strength due to the material scatter. As soon as the ultimate tensile strength of the weakest element is reached, it suddenly fails, and the force is absorbed by the remaining elements. Nevertheless, a redistribution can only take place if the remaining fibre strands have sufficient residual load-bearing capacity, which is only possible with a high number of fibre strands, or a very large spread of the ultimate tensile strength. Considering a system of *n* identical fibre strands, the ultimate tensile strengths *X*_i_ following a cumulative distribution function $F_{X} \left( {\it{x}} \right)$, the ultimate tensile strength *R* of the system can be described as^41^:

15 $$R = \max \left( {n \cdot \widehat{X}_{1} , \left( {n - 1} \right) \cdot \widehat{X}_{2} , \ldots , \, \widehat{X}_{n} } \right)$$

with $\widehat{X}_{1} , \ldots , \widehat{X}_{n}$ being the ultimate tensile strength of the individual strand sorted in ascending order by size. As a safe side approximation, it can be assumed that the weakest link governs the failure mechanism. Thus, a parallel connection can be compared to the behaviour of a series connection due to the nearly ideal brittle behaviour of the components. Consequently, the calculation of the cumulative distribution function of the minima $F_{{M_{n} }} \left( {\it{x}} \right)$ can be approximately calculated with Eq. (6).

#### Comparison of the experimental and theoretical investigations

**Figure 10:**
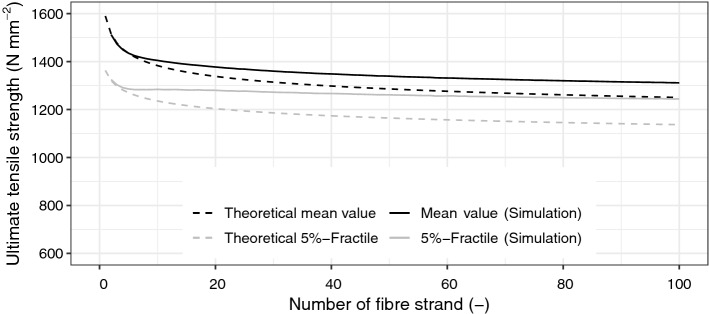
Ultimate tensile strength depending on the number of fibre strands.

Based on the above-described considerations, the experimental and theoretical investigations are compared and discussed. To this, a chain system of fibre strands was considered under the assumption that redistribution of stresses cannot occur when the weakest fibre strand fails, as described above. Then, the theoretical mean value of the ultimate tensile strength as well as the characteristic value the ultimate tensile strengths (5%-Fractile) were assessed by considering the extreme value distribution type I (Gumbel distribution). Then, these values were compared to the results determined through simulations. In this evaluation, it was assumed that each fibre strand of a chain is normally distributed. The values described in Sect. “Material parameters for the design approach” were used to characterise the ultimate tensile strength, where the mean ultimate tensile strength is ${ }\mu_{{\text{X}}} \approx \overline{x}_{{\text{X}}} = 1\;590\;{\text{N}}\;{\text{mm}}^{ - 2}$ and the standard deviation is $\sigma_{{\text{X}}} \approx s_{{\text{X}}} = 138\;{\text{N}}\;{\text{mm}}^{ - 2}$. By using the principles of a direct Monte-Carlo simulation, 50 000 simulations were performed in the statistical software *R*^42^. Furthermore, a theoretical calculation was also performed by assessing the expected value through Eq. (7). The results are illustrated in Fig. 10 and summarised in Table 2.

**Table 2:** Ultimate tensile strength: Comparison of the simulations and the theoretical investigations.

No. fibre strands	Expected value (N mm^−2^) (i.e., mean value)			5%-Fractile value (N mm^−2^)		
	simulation	Gumbel distribution	Difference (%)	Simulation	Gumbel distribution	Difference (%)
5	1 435	1 434	− 0.07	1 281	1 270	− 0.87
10	1 404	1 383	− 1.55	1 282	1 236	− 3.71
25	1 367	1 325	− 3.21	1 272	1 194	− 6.57
50	1 339	1 286	− 4.13	1 258	1 165	− 7.96
75	1 323	1 265	− 4.55	1 247	1 148	− 8.63
100	1 311	1 250	− 4.90	1 241	1 137	− 9.10

The results of the simulation confirm that the mean ultimate tensile strength decreases as the number of fibre strands increases, where the curves tend to flatten. Consequently, the standard deviation and the coefficient of variation also decrease with an increasing number of fibre strands. For an increasing number of fibre strands, the extreme value distribution approximated by a Gumbel distribution loses expression (i.e., decreases at a very slow pace). The results also show that the differences between the simulated values and the mathematical approximation through a Gumbel distribution can go up to around 9%, which can be explained by the fact that a Gumbel distribution does not consider a redistribution of stresses after the failure of the first fibre strand. These results confirm that a Gumbel approximation is on the safe side.

### Summary of the tests

The above-described results seem to support the assumption that standardised tensile test performed for a single fibre strand with a length of 160 mm is considered sufficient to determine all the statistical parameters needed for the design model of a textile reinforcement – and ultimately, for further reliability assessments. To this, the ultimate tensile strength can be characterised by a Gumbel distribution. This fitting must be done for a reasonable number of fibre strands and for a specific expected value $\mu_{{\text{X}}}$ and specific standard deviation $\sigma_{{\text{X}}}$. It is relevant to mention that such number of fibre strands is complex to define with precision. However, the authors believe that assuming at least 50 fibre strands is a safe assumption since it is not conceivable that the redistribution of stresses takes place beyond such number. In fact, for larger numbers the fibre strands will be distant from each other to allow for a redistribution. In its turn, the modulus of elasticity can be described by a Normal distribution.

## Usability of the results

**Figure 11:**
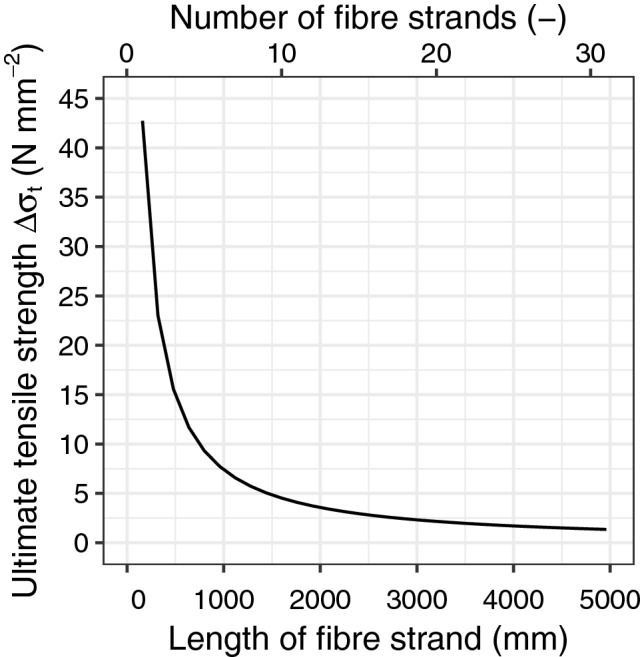
Margin of the 5%-Fractile values of n and (n-1)-fibre strands^15^.

The designed value of the tensile strength $f_{{{\text{td}}}}$ is the basis for the structural calculations with bending and shear load, which are described in^22,43–49^. As it has been discussed in this paper, the ultimate tensile strength depends on the length and number of fibre strands, which means that the material parameters determined for individual fibre strands cannot be directly transferred to the behaviour of a component. Since the strain length will be greater than 160 mm and the number of fibre strands will be more than one, a conversion must take place. Such conversion raises the question of which number or length of fibre strand is the most reasonable to represent a realistic reinforced concrete element. A possible answer could be the 5%-Fractile values since these so-called characteristic tensile strength of the reinforcement $f_{{{\text{tk}}}}$ form the basis for the design values of the tensile strength $f_{{{\text{td}}}}$ (Eq. 14 and Fig. 3b). Considering that the 5%-Fractile needs to be used for the design, the problem is not as pronounced, as it can be seen in Fig. 11, where the absolute differences of the textile stresses of the 5%-Fractile for the different lengths and numbers of fibre strands are illustrated. The difference is generated from the 5%-Fractile values of the ultimate tensile strength of *n* and (*n* − 1) fibre strands.

Figure 11 also indicates that for a small number of fibre strands, the 5%-Fractile of the tensile strength is strongly affected by the number of fibre strands. Whereas, from around five strands, the curve flattens out sharply and the difference between the characteristic values becomes gradually smaller. From a length of 1 × 600 mm, which corresponds to ten strands, the gradient is almost constant. This means that the 5%-Fractile value is only slightly higher for ten fibre strands than for eleven. In the case of the AR-glass examined, the value is 4.5 N mm^−2^, which corresponds to just 2.8‰ of the mean ultimate tensile strength. Therefore, it is recommended, to use a reasonable number of fibre strands for the determination of the characteristic value, which is in the area, where the curve slope of the 5%-Fractile differences becomes almost constant.

As explained before, the standardised tensile test on an individual fibre strand needs to be carried out, and then, the ultimate tensile strength must be adjusted by using the extreme value theory through Eqs. (6) and (7). With this approximation, the mean value $f_{{{\text{tm}}}}$, the characteristic value $f_{{{\text{tk}}}} ,$ and finally, the design value $f_{{{\text{td}}}}$ can be determined.

The design strain $\varepsilon_{{{\text{td}}}}$ is also required for the design model. To this, it is sufficient to measure the textile tension and divide it by the modulus of elasticity (Eq. 3). The tests showed that the modulus of elasticity is not influenced by the number of fibre strands. The mean value from the standardised test on a single fibre strand can be used as an appropriate modulus of elasticity.

## Conclusion

The results show that the standardised tensile test is sufficient to determine all the statistical data and material parameters necessary for the structural design of concrete components with textile reinforcement impregnated with epoxy resin. To this end, only the measurements of the ultimate tensile strength and the modulus of elasticity of a fibre strand, cut out of the textile grid are needed.

The tests show that a fibre strand, when subjected to tensile stress, has a linear-elastic behaviour until it fails. The results also indicate that the ultimate tensile strength depends on both the length and number of fibre strands. With an increasing length and number of fibre strands, the expected value and the scatter of the ultimate tensile strength decrease non-linearly. As soon as a certain length and number is exceeded, the characteristic ultimate tensile strength is hardly influenced. With the help of extreme value theory, the statistical values can be calculated for any length and number of fibre strands. To simplify the calculation, the extreme value distribution can be approximated by a Gumbel distribution. The approximation by a Normal distribution is not recommended. The main advantage of the described approach is that only the results of the standardised tensile test on an individual fibre strand are needed. Afterwards, a reliability assessment calculation can be carried out to derive an appropriate partial safety factor for design purposes.

Among the future challenges, the most prevailing is the improvement of the current theoretical model adopted in this study. The extreme value distribution was selected to describe the weakest ultimate tensile strength; however, the mathematical model adopted does not consider the possibility to redistribute the load. A more robust mathematical model shall be considered in future work. Focus shall be also placed on investigating a possible correlation between the modulus of elasticity and the ultimate tensile strength.
